# Short synthesis of the common trisaccharide core of kankanose and kankanoside isolated from *Cistanche tubulosa*

**DOI:** 10.3762/bjoc.9.80

**Published:** 2013-04-11

**Authors:** Goutam Guchhait, Anup Kumar Misra

**Affiliations:** 1 Bose Institute, Division of Molecular Medicine, P-1/12, C.I.T. Scheme VII M, Kolkata 700054, India

**Keywords:** *Cistanche tubulosa*, glycosylation, kankanoside, synthesis, trisaccharide

## Abstract

A short synthetic approach was developed for the synthesis of a common trisaccharide core found in kankanose, kankanoside F, H_1_, H_2_, and I isolated from the medicinally active plant *Cistanche tubulosa*. All glycosylations were carried out under nonmetallic reaction conditions. Yields were very good in all intermediate steps.

## Introduction

**Figure 1:**
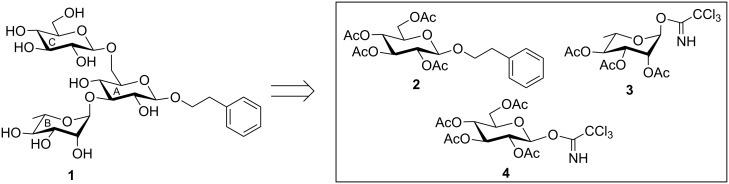
Structure of the synthesized trisaccharide core found in kankanose, kankanoside F, H_1_, H_2_ and I isolated from *Cistanche tubulosa.*

*Cistanche tubulosa* (*C. tubulosa*), an *Orobanchaceae* parasitic plant found in Africa, Asia and Arabia, has been traditionally used as folk medicine and tonic for the treatment of blood-circulation-related disorders, impotence, sterility and body weakness [1–3]. A significant number of bioactive compounds have been isolated from *C. tubulosa* and have shown promising medicinal activity such as hepatoprotective and vasorelaxant activities [4–6]. Most of the compounds isolated from *C. tubulosa* and related species are phenylethyl oligosaccharides, iridoids, terpenes and lignans [4–7]. Recently, Yoshikawa et al. isolated and characterized a significant number of phenylethyl oligosaccharides, which include kankanose, kankanoside F, H_1_, H_2_, I, etc. [4–5]. Since *C. tubulosa* has been used in the folk medicine for several years, it is beneficial to find out the biological activities of the individual compounds present in the *C. tubulosa* extracts. In order to establish the detailed medicinal potential of individual components, it is essential to have higher quantities of the compounds, which are difficult to isolate from the plant source. Therefore, development of concise chemical synthetic strategies would be the best option to gain access to these compounds on a large scale. A few reports are available in the literature for the synthesis of phenylethyl oligosaccharides [8–9]. In this context, we developed a synthetic strategy for the synthesis of the common trisaccharide core of kankanose, kankanoside F, H_1_, H_2_ and I isolated from *C. tubulosa* thereby exploiting newly developed regio- and stereoselective glycosylation conditions (Figure 1). This straightforward synthetic strategy employs a minimum number of steps.

## Results and Discussion

The target trisaccharide **1** in the form of its 2-phenylethyl glycoside was synthesized from the suitably functionalized monosaccharide derivatives **2** [10], **3** [11], and **4** [12], which were prepared from the commercially available reducing sugars (Figure 1). The key features of this synthetic strategy are (a) the application of two regioselective glycosylations by using glycosyl acceptors with two hydroxy groups; (b) application of molecular iodine to the functional group transformations [13]; and (c) activation of the glycosyl trichloroacetimidate derivative by using nitrosyl tetrafluoroborate (NOBF_4_) [14].

**Scheme 1:**
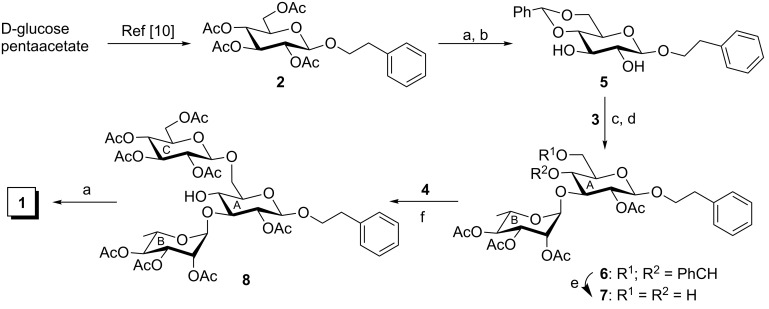
Reagents: (a) 0.1 M CH_3_ONa, CH_3_OH, room temperature, 3 h, 98% for compound **5**, 94% for compound **1**; (b) PhCH(OCH_3_)_2_, I_2_, CH_3_CN, room temperature, 1.5 h, 86%; (c) NOBF_4_, CH_2_Cl_2_, −20 °C, 20 min; (d) acetic anhydride, room temperature, 1 h, 76% in two steps; (e) Et_3_SiH, 10% Pd/C, CH_3_OH/CH_2_Cl_2_ (1:1, v/v), room temperature, 30 min, 80%; (f) NOBF_4_, CH_2_Cl_2_, −35 °C, 30 min, 71%.

The treatment of D-glucose pentaacetate with 2-phenylethanol in the presence of borontrifluoride diethyl etherate furnished 2-phenylethyl 2,3,4,6-tetra-*O*-acetyl-β-D-glucopyranoside (**2**) in 84% yield [10]. Saponification of compound **2** by using 0.1 M sodium methoxide in methanol followed by benzylidene acetal formation by using benzaldehyde dimethylacetal in the presence of molecular iodine [13] furnished compound **5** in 86% yield. Regioselective 3-*O*-glycosylation of compound **5** with L-rhamnose derived trichloroacetimidate derivative **3** [11] in the presence of NOBF_4_ [14] followed by acetylation in the same pot furnished disaccharide derivative **6** in 76% yield. In this case, NOBF_4_ acts as a promoter for the activation of the glycosyl trichloroacetimidate derivative as well as the acetylation of the sugar derivative with acetic anhydride. The formation of compound **6** was confirmed by its spectral analysis [signals at δ 5.44 (s, PhC*H*), 4.79 (br s, H-1_B_), 4.37 (d, *J* = 7.5 Hz, H-1_A_ in the ^1^H NMR and at δ 101.9 (Ph*C*H), 101.3 (C-1_A_), 97.4 (C-1_B_) in the ^13^C NMR spectra]. Removal of the benzylidene acetal group under neutral conditions by using triethylsilane and Pd/C [15] resulted in the formation of disaccharide diol **7** in 80% yield. NOBF_4_ catalyzed regio- and stereoselective 6-*O*-glycosylation of compound **7** with D-glucose-derived trichloroacetimidate derivative **4** [12] furnished trisaccharide derivative **8** in 71% yield, which was confirmed by the spectral analysis [signals at δ 4.75 (br s, H-1_B_), 4.54 (d, *J* = 8.0 Hz, H-1_C_), 4.22 (d, *J* = 8.0 Hz, H-1_A_) in the ^1^H NMR and at δ 100.8 (C-1_A_), 100.5 (C-1_C_), 98.8 (C-1_B_) in the ^13^C NMR spectra]. Saponification of compound **8** by using 0.1 M sodium methoxide in methanol furnished compound **1** in 94% yield. Spectral analysis of compound **1** unambiguously confirmed its formation [signals at δ 5.16 (br s, H-1_B_), 4.37 (d, *J* = 7.5 Hz, H-1_A_), 4.32 (d, *J* = 8.0 Hz, H-1_C_) in the ^1^H NMR and at δ 103.5 (C-1_A_), 102.9 (C-1_C_), 101.3 (C-1_B_) in the ^13^C NMR spectra] (Scheme 1).

## Conclusion

In summary, a straightforward synthetic strategy was developed for the prompt synthesis of a common trisaccharide core of the kankanose, kankanoside F, H_1_, H_2_ and I isolated from the extract of *C. tubulosa.* All the steps are high yielding, and glycosylations were highly regio- and stereoselective. Because of the simplicity of the synthetic strategy, it can be applied in a scaled-up preparation.

## Experimental

### General methods

All reactions were monitored by thin-layer chromatography with silica-gel-coated TLC plates. The spots on TLC were visualized by warming ceric sulfate (2% Ce(SO_4_)_2_ in 2 N H_2_SO_4_) sprayed plates on a hot plate. Silica gel 230–400 mesh was used for column chromatography. ^1^H and ^13^C NMR spectra were recorded on a Bruker Avance 500 MHz by using CDCl_3_ as the solvent and TMS as the internal reference unless stated otherwise. Chemical shifts δ are expressed in parts per million (ppm). ESIMS were recorded on a Micromass mass spectrometer. Optical rotations were recorded in a Jasco P-2000 spectrometer. Commercially available grades of organic solvents of adequate purity were used in all reactions.

**2-Phenylethyl 4,6-*O*-benzylidene-β-D-glucopyranoside (5)**: A solution of compound **2** (5.0 g, 11.05 mmol) in 0.1 M CH_3_ONa in CH_3_OH (70 mL) was stirred at room temperature for 3 h and was neutralized with Amberlite IR-120 (H^+^) resin. The reaction mixture was filtered and concentrated under reduced pressure. To a solution of the deacetylated product (3.1 g) in CH_3_CN (10 mL) was added benzaldehyde dimethylacetal (2.5 mL, 16.65 mmol) followed by molecular iodine (0.3 g, 1.18 mmol), and the reaction mixture was stirred at room temperature for 1.5 h. The reaction mixture was evaporated and co-evaporated with toluene (3 × 20 mL) under reduced pressure to give the crude product, which was purified over SiO_2_ by using hexane/EtOAc (2:1) as an eluant to give pure compound **5** (3.5 g, 86%). White solid; mp 158–160 °C (EtOH); [α]_D_^25^ −16 (*c* 1.2, CHCl_3_); ^1^H NMR (500 MHz, CDCl_3_) δ 7.48–7.21 (m, 10H, Ar-H), 5.50 (s, 1H, PhC*H*), 4.35 (d, *J* = 7.7 Hz, 1H, H-1), 4.32 (dd, *J* = 10.5, 4.9 Hz, 1H, H-6_a_), 4.17–4.12 (m, 1H, C*H*_2_), 3.78–3.72 (m, 3H, H-3, H-6_b_, C*H*_2_), 3.52 (t, *J* = 9.3 Hz, 1H, H-4), 3.47–3.39 (m, 2H, H-2, H-5), 2.99–2.93 (m, 2H, C*H*_2_), 2.90 (br s, 1H, O*H*), 2.54 (br s, 1H, O*H*); ^13^C NMR (125 MHz, CDCl_3_) δ 138.1–126.2 (Ar-C), 103.3 (C-1), 101.9 (Ph*C*H), 80.5 (C-5), 74.5 (C-3), 72.9 (C-4), 71.1 (C-2), 68.6 (*C*H_2_), 66.4 (C-6), 36.1 (*C*H_2_); ESIMS: 395.1 [M + Na]^+^; Anal. calcd for C_21_H_24_O_6_: C, 67.73; H, 6.50; found: C, 67.57; H, 6.65.

**2-Phenylethyl (2,3,4-tri-*O*-acetyl-α-L-rhamnopyranosyl)-(1→3)-2-*O*-acetyl-4,6-*O*-benzylidene-β-D-glucopyranoside (6)**: A solution of compound **5** (3.0 g, 8.06 mmol) and compound **3** (3.6 g, 8.28 mmol) in anhydrous CH_2_Cl_2_ (15 mL) was cooled to −20 °C under argon. To the cooled reaction mixture was added NOBF_4_ (1.0 g, 8.56 mmol), and the reaction mixture was stirred at the same temperature for 20 min. After consumption of the starting material (TLC; hexane/EtOAc 4:1), acetic anhydride (3 mL) was added to the reaction mixture, and the mixture was stirred at room temperature for 1 h. The reaction mixture was diluted with CH_2_Cl_2_ (100 mL) and the organic layer was washed with satd. NaHCO_3_ and water, dried (Na_2_SO_4_) and concentrated to a crude product, which was purified over SiO_2_ by using hexane/EtOAc (3:1) as an eluant to give the pure product **6** (4.2 g, 76%). Yellow oil; [α]_D_^25^ −60 (*c* 1.2, CHCl_3_); ^1^H NMR (500 MHz, CDCl_3_) δ 7.39–7.11 (m, 10H, Ar-H), 5.44 (s, 1H, PhC*H*), 5.23 (dd, *J* = 10.0, 3.5 Hz, 1H, H-3_B_), 4.97 (t, *J* = 8.0 Hz, 1H, H-2_A_), 4.89 (br s, 1H, H-2_B_), 4.85 (t, *J* = 10.0 Hz, 1H, H-4_B_), 4.79 (br s, 1H, H-1_B_), 4.37 (d, *J* = 7.5 Hz, 1H, H-1_A_), 4.27 (dd, *J* = 10.5, 5.0 Hz, 1H, H-6_aA_), 4.04–3.97 (m, 2H, H-5_A_, C*H*_2_), 3.78 (t, *J* = 9.5 Hz, 1H, H-3_A_), 3.69 (t, *J* = 10.0 Hz, 1H, H-6_bA_), 3.60–3.54 (m, 2H, H-4_A_, C*H*_2_), 3.39–3.36 (m, 1H, H-5_B_), 2.81–2.78 (m, 2H, C*H*_2_), 2.01, 1.91, 1.89, 1.88 (4 s, 12H, 4 COC*H*_3_), 0.59 (d, *J* = 6.0 Hz, 3H, CC*H*_3_); ^13^C NMR (125 MHz, CDCl_3_) δ 170.0, 169.9, 169.8, 169.4 (4 *C*OCH_3_), 138.5–126.3 (Ar-C), 101.9 (Ph*C*H), 101.3 (C-1_A_), 97.4 (C-1_B_), 79.0 (C-4_A_), 76.8 (C-3_A_), 73.3 (C-2_A_), 71.3 (C-4_B_), 70.6 (C-2_B_), 70.5 (*C*H_2_), 68.7 (C-6_A_), 68.4 (C-3_B_), 66.6 (C-5_B_), 66.2 (C-5_A_), 36.0 (*C*H_2_), 20.9, 20.8, 20.7, 20.6 (4 CO*C*H_3_), 16.5 (*C*H_3_); ESIMS: 709.2 [M + Na]^+^; Anal. calcd for C_35_H_42_O_14_: C, 61.22; H, 6.16; found: C, 61.05; H, 6.35.

**2-Phenylethyl (2,3,4-tri-*O*-acetyl-α-L-rhamnopyranosyl)-(1→3)-2-*O*-acetyl-β-D-glucopyranoside (7)**: To a stirred solution of compound **6** (4.0 g, 5.82 mmol) and 10% Pd/C (0.5 g) in CH_3_OH/CH_2_Cl_2_ (15 mL, 1:1, v/v) was added Et_3_SiH (2.8 mL, 17.53 mmol) dropwise, and the reaction mixture was stirred for 30 min at room temperature. The reaction mixture was filtered through a Celite^®^ bed, and the filtering bed was washed with CH_2_Cl_2_ (50 mL). The combined filtrate was concentrated under reduced pressure to give the crude product, which was purified over SiO_2_ by using hexane/EtOAc (1:1) as an eluant to give pure compound **7** (2.8 g, 80%). Yellow oil; [α]_D_^25^ −30 (*c* 1.2, CHCl_3_); ^1^H NMR (500 MHz, CDCl_3_) δ 7.28–7.18 (m, 5H, Ar-H), 5.23 (dd, *J* = 10.0, 3.5 Hz, 1H, H-3_B_), 5.10–5.09 (m, 1H, H-2_B_), 5.05 (t, *J* = 10.0 Hz, 1H, H-4_B_), 4.94 (t, *J* = 8.0 Hz, 1H, H-2_A_), 4.87 (d, *J* = 1.8 Hz, 1H, H-1_B_), 4.41 (d, *J* = 8.0 Hz, 1H, H-1_A_), 4.16–4.06 (m, 2H, C*H*_2_), 3.92–3.86 (m, 1H, H-6_aA_), 3.82–3.78 (m, 1H, H-6_bA_), 3.69–3.61 (m, 2H, H-3_A_, H-5_B_), 3.58 (t, *J* = 9.0 Hz, 1H, H-4_A_), 3.54–3.31 (m, 1H, H-5_A_), 2.91–2.83 (m, 2H, C*H*_2_), 2.13, 2.04, 2.01, 1.98 (4 s, 12H, 4 COC*H*_3_), 1.21 (d, *J* = 6.0 Hz, 3H, C*H*_3_); ^13^C NMR (125 MHZ, CDCl_3_) δ 170.0, 169.9, 169.7, 169.5 (4 *C*OCH_3_), 138.5–126.3 (Ar-C), 100.7 (C-1_A_), 98.8 (C-1_B_), 84.8 (C-4_A_), 75.2 (C-3_A_), 71.4 (C-2_A_), 70.7 (C-4_B_), 70.4 (C-2_B_), 69.9 (2C, C-5_B_, *C*H_2_), 68.6 (C-3_B_), 67.7 (C-5_A_), 62.2 (C-6_A_), 36.0 (*C*H_2_), 20.9, 20.8, 20.7, 20.6 (4 CO*C*H_3_), 17.4 (*C*H_3_); ESIMS: 621.2 [M + Na]^+^; Anal. calcd for C_28_H_38_O_14_: C, 56.18; H, 6.40; found: C, 56.05; H, 6.55.

**2-Phenylethyl (2,3,4-tri-*O*-acetyl-α-L-rhamnopyranosyl)-(1→3)-[2,3,4,6-tetra-*O*-acetyl-β-D-glucopyranosyl)-(1→6)]-2-*O*-acetyl-β-D-glucopyranoside (8):** A solution of compound **4** (1.7 g, 3.45 mmol) and compound **7** (2.0 g, 3.34 mmol) in anhydrous CH_2_Cl_2_ (20 mL) was cooled to −35 °C under argon. To the cooled reaction mixture was added NOBF_4_ (410 mg, 3.51 mmol), and the reaction mixture was stirred at the same temperature for 30 min. The reaction mixture was diluted with CH_2_Cl_2_ (100 mL), and the organic layer was washed with satd. NaHCO_3_ and water, dried (Na_2_SO_4_), and concentrated to a crude product, which was purified over SiO_2_ by using hexane/EtOAc (3:1) as an eluant to give the pure product **8** (2.2 g, 71%). Yellow oil; [α]_D_^25^ −32 (*c* 1.2, CHCl_3_); ^1^H NMR (500 MHz, CDCl_3_) δ 7.17–7.07 (m, 5H, Ar-H), 5.12 (dd, *J* = 10.0, 3.5 Hz, 1H, H-3_B_), 5.07 (t, *J* = 10.0 Hz, 1H, H-3_C_), 4.99–4.93 (m, 3H, H-2_B_, H-4_B_, H-4_C_), 4.89 (t, *J* = 10.0 Hz, 1H, H-2_C_), 4.82 (t, *J* = 10.0 Hz, 1H, H-2_A_), 4.75 (br s, 1H, H-1_B_), 4.54 (d, *J* = 8.0 Hz, 1H, H-1_C_), 4.22 (d, *J* = 8.0 Hz, 1H, H-1_A_), 4.16 (dd, *J* = 12.0, 5.0 Hz, 1H, H-6_aC_), 4.07–3.96 (m, H-6_aA_, H-6_bC_, C*H*_2_), 3.69–3.65 (m, 1H, H-6_bA_), 3.60–3.56 (m, 1H, H-5_A_), 3.55–3.48 (m, 1H, C*H*_2_), 3.43 (t, *J* = 10.0 Hz, 1H, H-3_A_), 3.42–3.35 (m, 1H, H-5_C_), 3.33–3.31 (m, 1H, H-5_B_), 2.77–2.74 (m, 2H, C*H*_2_), 2.08, 2.07, 2.01, 2.00, 1.99, 1.96, 1.94, 1.90 (8 s, 24H, 8 COC*H*_3_), 1.10 (d, *J* = 6.0 Hz, 3H, C*H*_3_); ^13^C NMR (125 MHz, CDCl_3_) δ 170.7, 170.2, 170.0, 169.9, 169.6, 169.4, 169.3, 169.2 (8 *C*OCH_3_), 138.6–126.2 (Ar-C), 100.8 (C-1_A_), 100.5 (C-1_C_), 98.8 (C-1_B_), 84.3 (C-5_C_), 74.8 (C-5_B_), 72.7 (C-3_C_), 71.9 (C-4_A_), 71.5 (C-2_B_), 71.1 (C-5_A_), 70.8 (C-2_C_), 70.3 (*C*H_2_), 70.2 (C-2_A_), 70.0 (C-3_A_), 68.7 (C-6_A_), 68.5 (C-4_C_), 68.2 (C-4_B_), 67.5 (C-3_B_), 61.7 (C-6_C_), 35.9 (*C*H_2_), 20.9, 20.8, 20.7 (3 C), 20.6 (3 C) (8 CO*C*H_3_), 17.3 (*C*H_3_); ESIMS: 951.3 [M + Na]^+^; Anal. calcd for C_42_H_56_O_23_: C, 54.31; H, 6.08; found: C, 54.18; H, 6.25.

**2-Phenylethyl (α-L-rhamnopyranosyl)-(1→3)-[β-D-glucopyranosyl)-(1→6)]-β-D-glucopyranoside (1):** A solution of compound **8** (2.0 g, 2.15 mmol) in 0.1 M CH_3_ONa in CH_3_OH (50 mL) was stirred at room temperature for 3 h and neutralized with Amberlite IR-120 (H^+^) resin. The reaction mixture was filtered and concentrated to give the crude product, which was purified over Sephadex^®^ LH-20 gel by using CH_3_OH/H_2_O (10:1) as an eluant to give pure compound **1** (1.2 g, 94%). White powder; [α]_D_^25^ −11 (*c* 1.2, CH_3_OH); ^1^H NMR (500 MHz, CD_3_OD) δ 7.26–7.16 (m, 5H, Ar-H), 5.16 (br s, 1H, H-1_B_), 4.37 (d, *J* = 7.5 Hz, 1H, H-1_A_), 4.32 (d, *J* = 8.0 Hz, 1H, H-1_C_), 4.15–4.13 (m, 1H, H-6_aC_), 4.10–4.04 (m, 1H, C*H*_2_), 4.03–3.98 (m, 1H, H-5_B_), 3.95–3.92 (m, 1H, H-2_B_), 3.88–3.82 (m, 1H, H-6_aA_), 3.81–3.73 (m, 2H, H-6_bC_, C*H*_2_), 3.70 (dd, *J* = 10.0, 3.5 Hz, 1H, H-3_B_), 3.68–3.64 (m, 1H, H-6_bA_), 3.50 (t, *J* = 10.0 Hz, 1H, H-4_A_), 3.48–3.44 (m, 2H, H-4_B_, H-5_A_), 3.39 (t, *J* = 10.0 Hz, 1H, H-4_C_), 3.30–3.35 (m, 4H, H-2_A_, H-3_A_, H-3_C_, H-5_C_), 3.21 (t, *J* = 9.0 Hz, 1H, H-2_C_), 2.94–2.90 (m, 2H, C*H*_2_), 1.25 (d, *J* = 6.0 Hz, 3H, C*H*_3_); ^13^C NMR (125 MHz, CD_3_OD) δ 138.6–125.8 (Ar-C), 103.5 (C-1_A_), 102.9 (C-1_C_), 101.3 (C-1_B_), 82.6 (C-4_A_), 76.6 (2C, C-3_A_, C-3_C_), 75.6 (C-5_A_), 74.3 (C-5_C_), 73.7 (C-2_C_), 72.6 (C-4_C_), 71.0 (C-4_B_), 70.9 (C-2_A_), 70.6 (*C*H_2_), 70.2 (C-2_B_), 68.6 (C-3_B_), 68.5 (C-5_B_), 68.3 (C-6_C_), 61.3 (C-6_A_), 35.8 (*C*H_2_), 16.5 (*C*H_3_); ESIMS: 615.2 [M + Na]^+^; Anal. calcd for C_26_H_40_O_15_: C, 52.70; H, 6.80; found: C, 52.56; H, 7.0.

## Supporting Information

File 1 ^1^H NMR and ^13^C NMR spectra of compounds **1, 2, 5, 6, 7** and **8**.
