# Supplementary material for: Short synthesis of the common trisaccharide core of kankanose and kankanoside isolated from Cistanche tubulosa
Source: Beilstein J Org Chem. 2013 Apr 11;9:705–9. doi: 10.3762/bjoc.9.80 (PMC3678509; doi:10.3762/bjoc.9.80)
Supplement: File 1 — 1H NMR and 13C NMR spectra of compounds 1, 2, 5, 6, 7 and 8. [file Beilstein_J_Org_Chem-09-705-s001.pdf]

Supporting Information

for

Short synthesis of the common trisaccharide core of kankanose and kankanoside isolated from *Cistanche tubulosa*

Goutam Guchhait and Anup Kumar Misra*[§]

Address: Bose Institute, Division of Molecular Medicine, P-1/12, C.I.T. Scheme VII M, Kolkata 700054, India

E-mail: Anup Kumar Misra - akmisra69@gmail.com

*Corresponding author

[§]Fax: 91-33-2355 3886

^{1}H NMR and ^{13}C NMR spectra of compounds 1, 2, 5, 6, 7 and 8

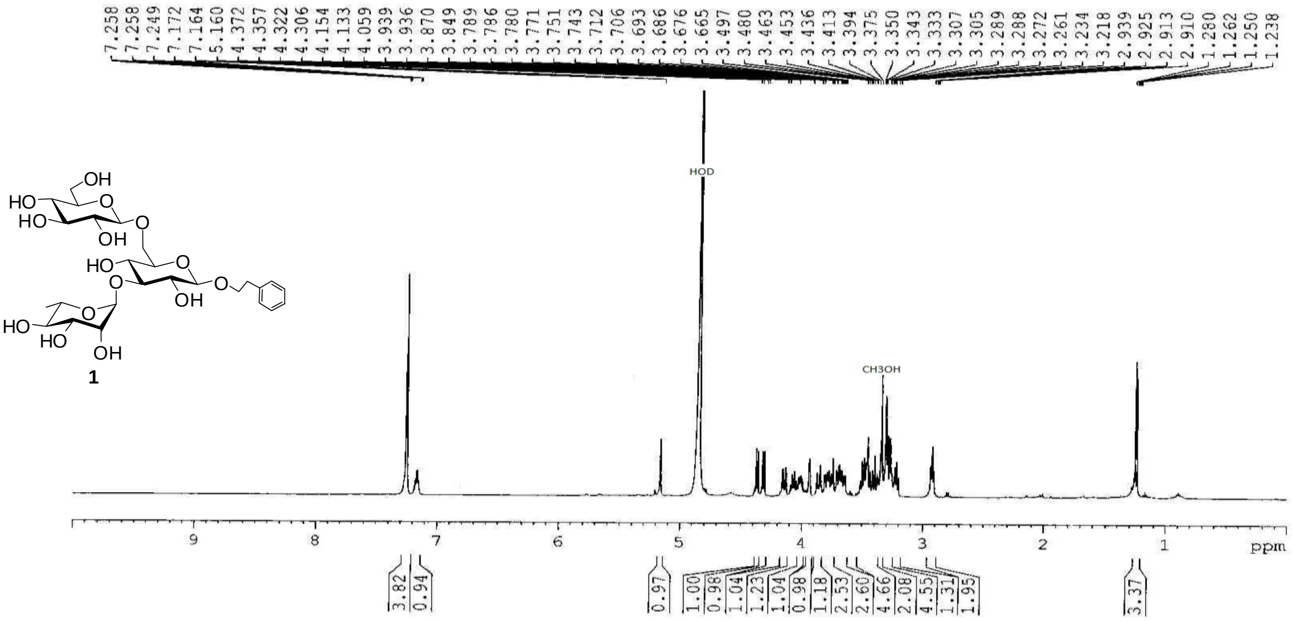
7.258 7.258 7.249 7.172 7.164 5.160 4.372 4.357 4.322 4.306 4.154 4.133 4.059 3.939 3.936 3.870 3.849 3.789 3.786 3.780 3.771 3.751 3.743 3.712 3.706 3.693 3.686 3.676 3.665 3.497 3.480 3.463 3.453 3.436 3.413 3.394 3.375 3.350 3.343 3.333 3.307 3.305 3.289 3.288 3.272 3.261 3.234 3.218 2.939 2.925 2.913 2.910 1.280 1.262 1.250 1.238
HOD
CH3OH
1
ppm
3.82 0.94 0.97 1.00 0.98 1.04 1.23 1.04 0.98 1.18 2.53 2.60 4.66 2.08 4.55 1.31 1.95 3.37

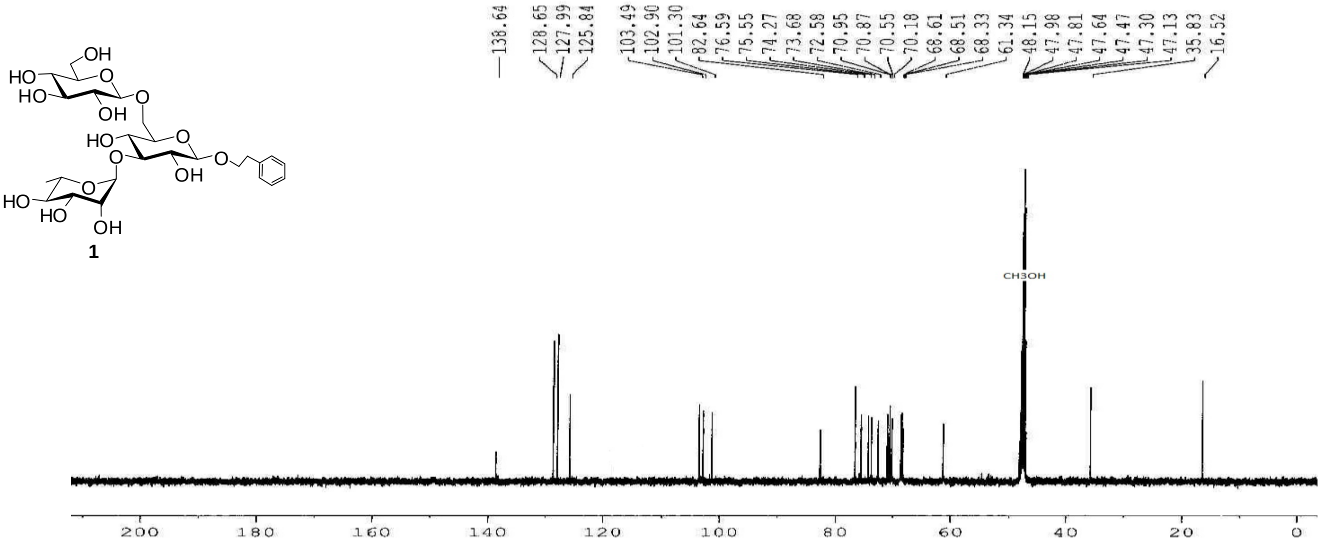
138.64 128.65 127.99 125.84 103.49 102.90 101.30 82.64 76.59 75.55 74.27 73.68 72.58 70.95 70.87 70.55 70.18 68.61 68.51 68.33 61.34 48.15 47.98 47.81 47.64 47.47 47.30 47.13 35.83 16.52
CH3OH
1

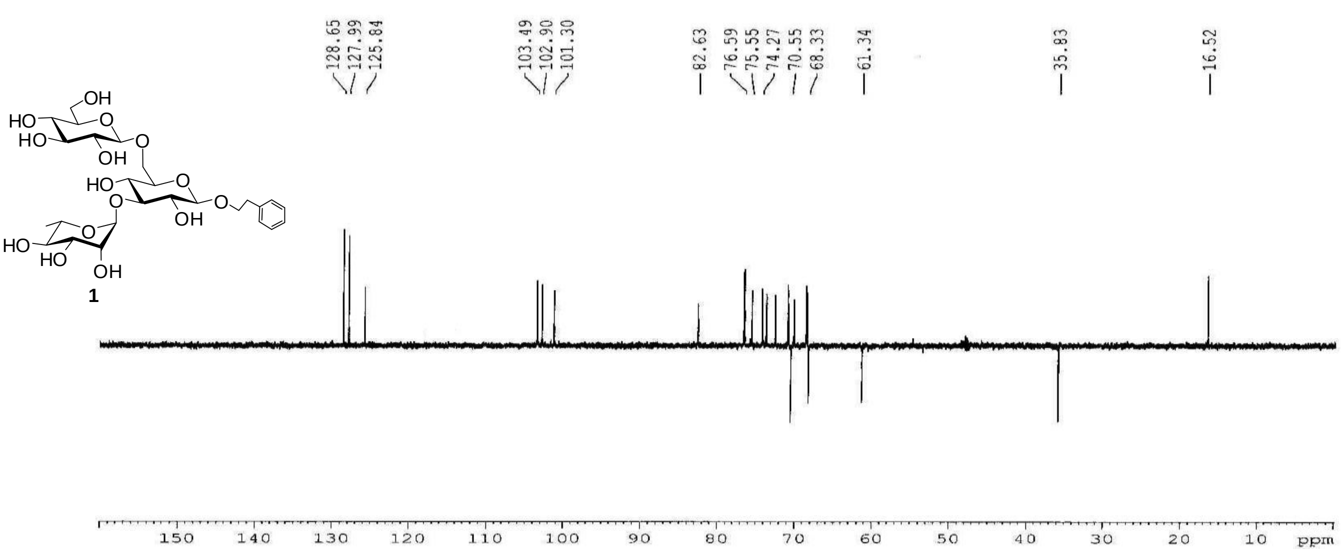
128.65 127.99 125.84 103.49 102.90 101.30 82.63 76.59 75.55 74.27 70.55 68.33 61.34 35.83 16.52
1
ppm

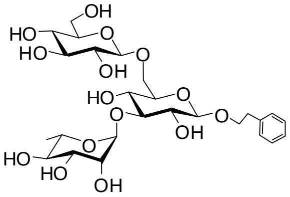
HO
HO
OH
OH
HO
O
O
O
OH
HO
HO
OH

1

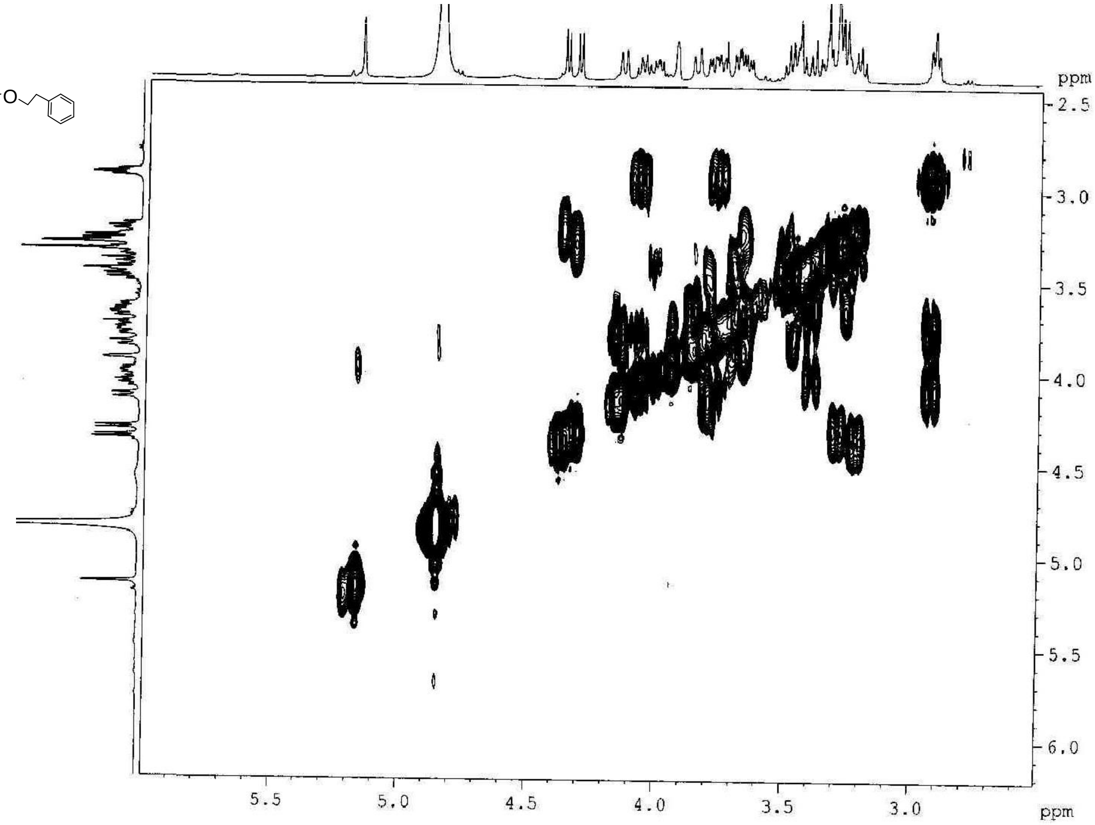
ppm
2.5
3.0
3.5
4.0
4.5
5.0
5.5
6.0
5.5
5.0
4.5
4.0
3.5
3.0
ppm

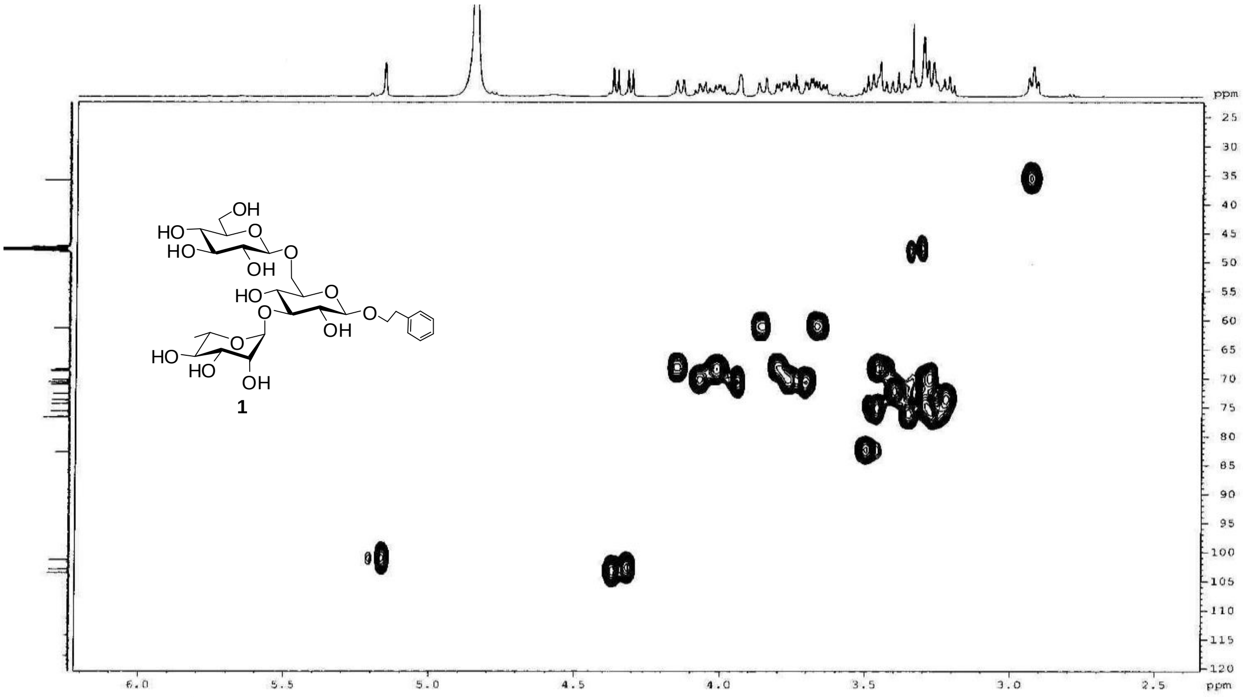
1
ppm
6.0
5.5
5.0
4.5
4.0
3.5
3.0
2.5
ppm

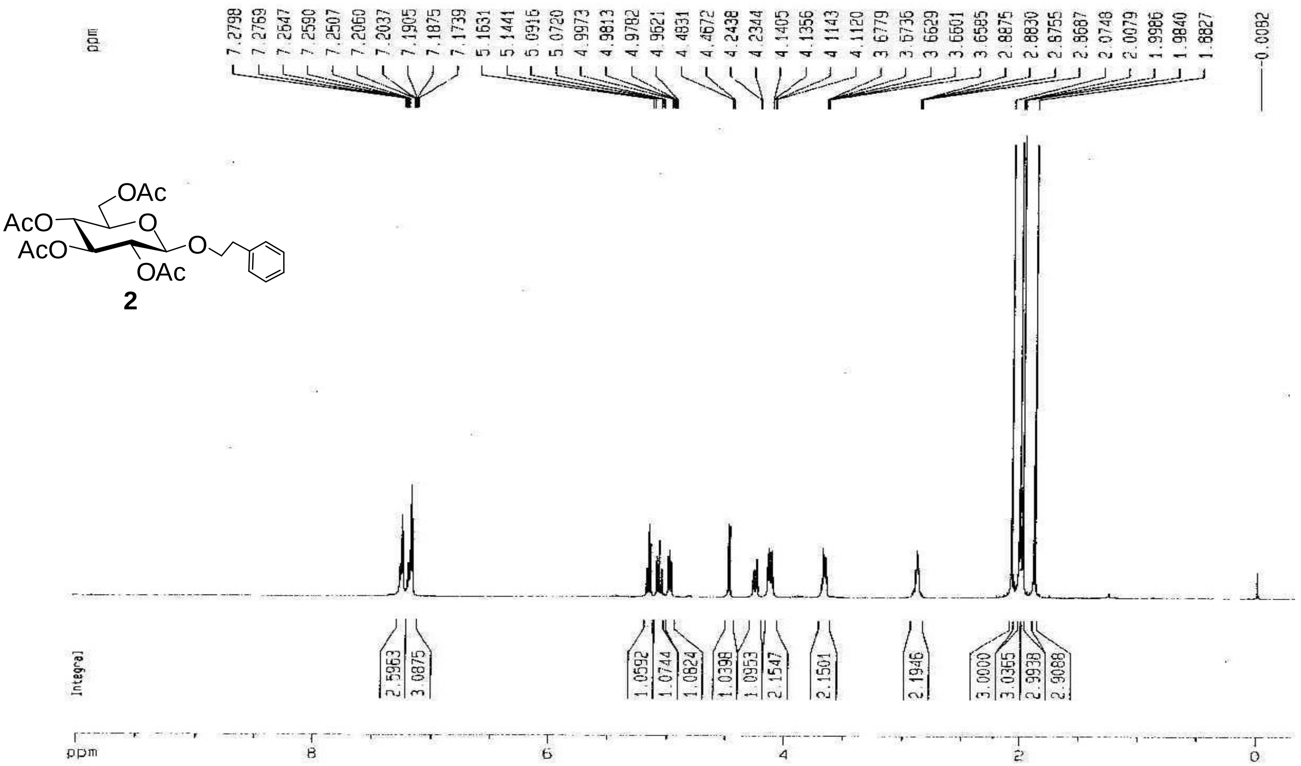
OAc
AcO
AcO
O
O
OAc
2
ppm
7.2798 7.2769 7.2647 7.2590 7.2507 7.2060 7.2037 7.1905 7.1875 7.1739 5.1631 5.1441 5.0916 5.0720 4.9973 4.9813 4.9782 4.9621 4.4831 4.4672 4.2438 4.2344 4.1405 4.1356 4.1143 4.1120 3.6779 3.6736 3.6629 3.6601 3.6585 2.8875 2.8830 2.8755 2.8687 2.0748 2.0079 1.9986 1.9840 1.8827 -0.0082
Integral
2.5963 3.0875 1.0592 1.0744 1.0824 1.0398 1.0953 2.1547 2.1501 2.1946 3.0000 3.0365 2.9938 2.9088
ppm
8
6
4
2
0

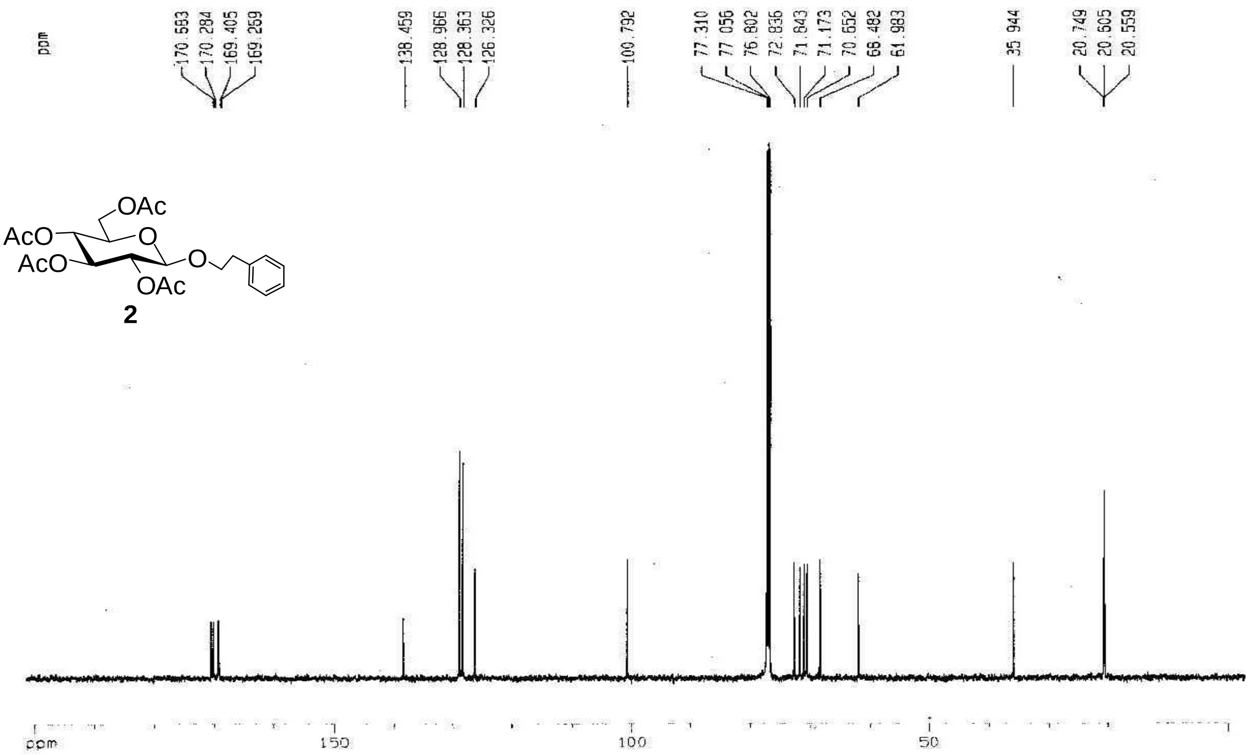
ppm
170.563 170.284 169.405 169.259 138.459 128.966 128.363 126.326 100.792 77.310 77.056 76.802 72.836 71.643 71.173 70.652 68.482 61.983 35.944 20.749 20.605 20.559
OAc
AcO
AcO
O
O
OAc
2
ppm
150
100
50

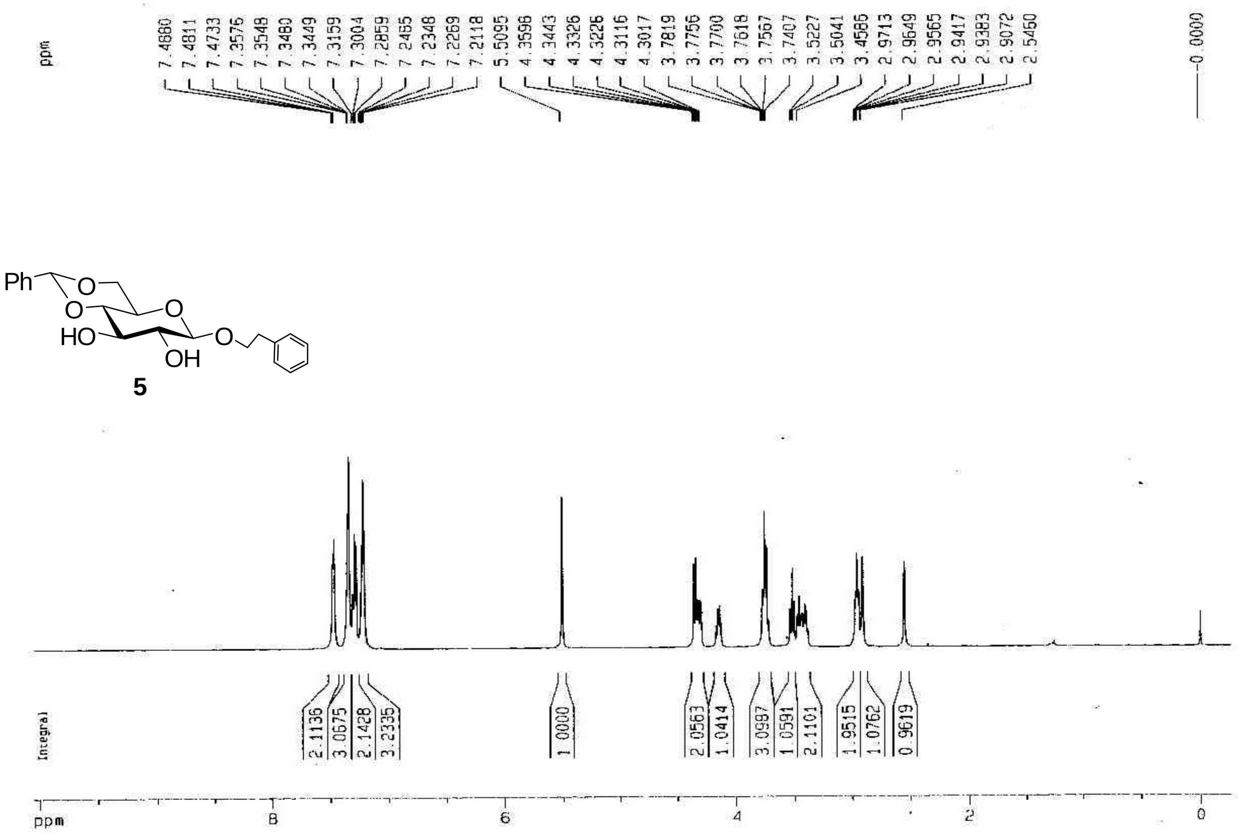

5
ppm
7.4860 7.4811 7.4733 7.3576 7.3548 7.3480 7.3449 7.3159 7.3004 7.2859 7.2465 7.2348 7.2269 7.2118
5.5095 4.3596 4.3443 4.3326 4.3226 4.3116 4.3017 3.7819 3.7756 3.7700 3.7618 3.7567 3.7407 3.5227 3.5041 3.4586 2.9713 2.9649 2.9565 2.9417 2.9383 2.9072 2.5450
0.0000
Integral
2.1136 3.0675 2.1428 3.2335 1.0000 2.0563 1.0414 3.0987 1.0591 2.1101 1.9515 1.0762 0.9619
ppm
8
6
4
2
0

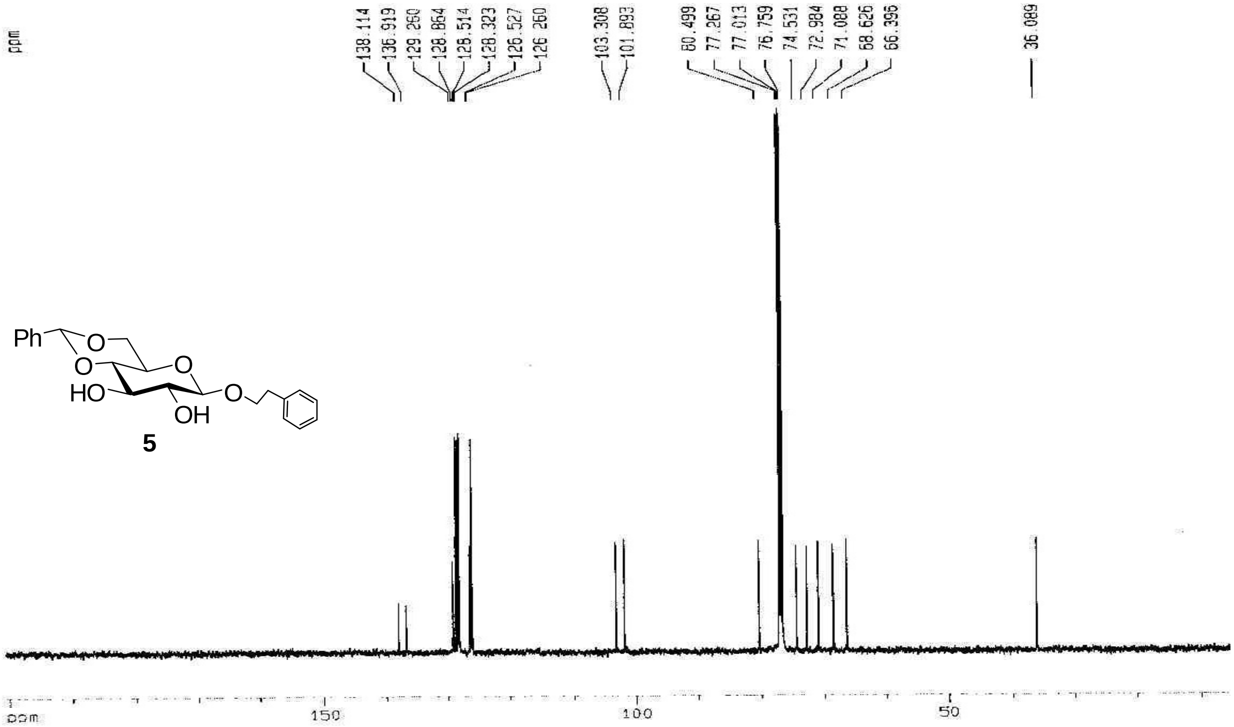
ppm
138.114 136.919 129.260 128.864 128.514 128.323 126.527 126.260
103.308 101.893
80.499 77.267 77.013 76.759 74.531 72.984 71.088 68.626 66.396
36.089
ppm
150
100
50

5

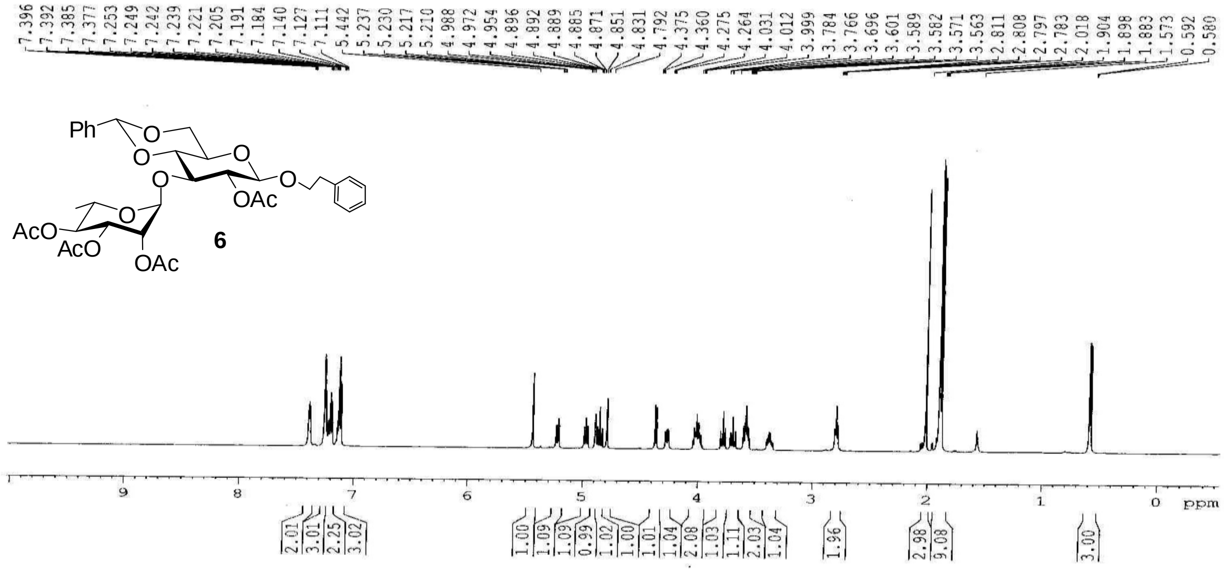

7.396 7.392 7.385 7.377 7.253 7.249 7.242 7.239 7.221 7.205 7.191 7.184 7.140 7.127 7.111 5.442 5.237 5.230 5.217 5.210 4.988 4.972 4.954 4.896 4.892 4.889 4.885 4.871 4.851 4.831 4.792 4.375 4.360 4.275 4.264 4.031 4.012 3.999 3.784 3.766 3.696 3.601 3.589 3.582 3.571 3.563 2.811 2.808 2.797 2.783 2.018 1.904 1.898 1.883 1.573 0.592 0.580
Ph
O
O
O
O
OAc
O
AcO
AcO
OAc
6
ppm
2.01 3.01 2.25 3.02 1.00 1.09 1.09 0.99 1.02 1.00 1.01 1.04 2.08 1.03 1.11 2.03 1.04 1.96 2.98 9.08 3.00

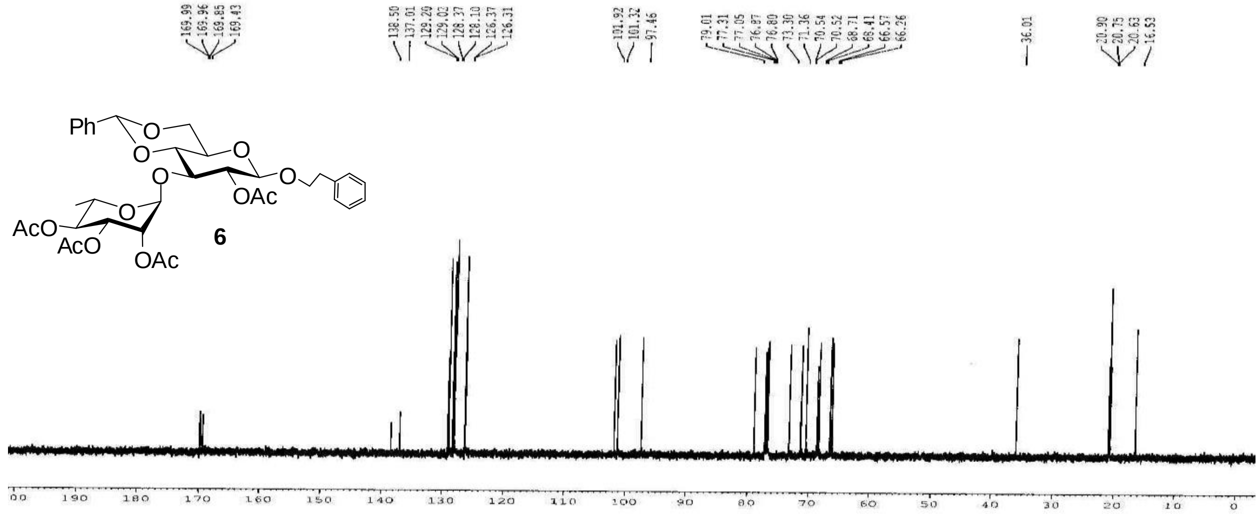

169.99 169.96 169.85 169.43 138.50 137.01 129.20 129.02 128.37 128.10 126.37 126.31 101.92 101.32 97.46 79.01 77.31 77.05 76.87 76.80 73.30 71.36 70.54 70.52 68.71 68.41 66.57 66.26 36.01 20.90 20.75 20.63 16.53
Ph
O
O
O
O
OAc
O
AcO
AcO
OAc
6

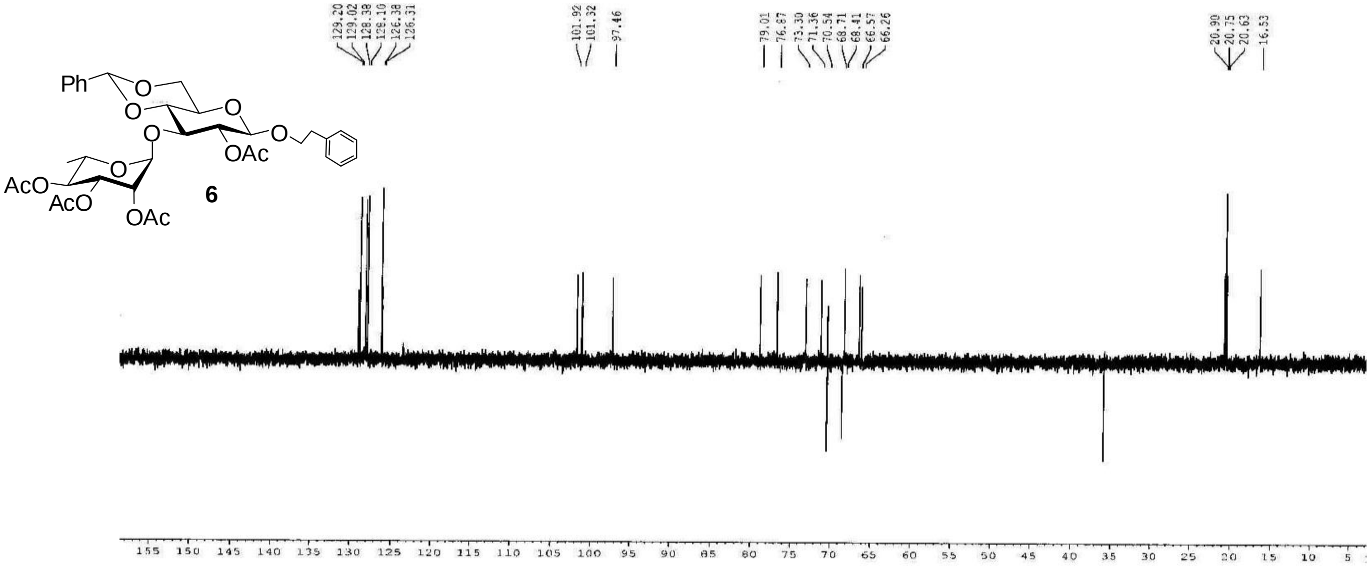

129.20 129.02 128.38 128.10 126.38 126.31 101.92 101.32 97.46 79.01 76.87 73.30 71.36 70.54 68.71 68.41 66.57 66.26 20.90 20.75 20.63 16.53
Ph
O
O
O
O
OAc
O
AcO
AcO
OAc
6

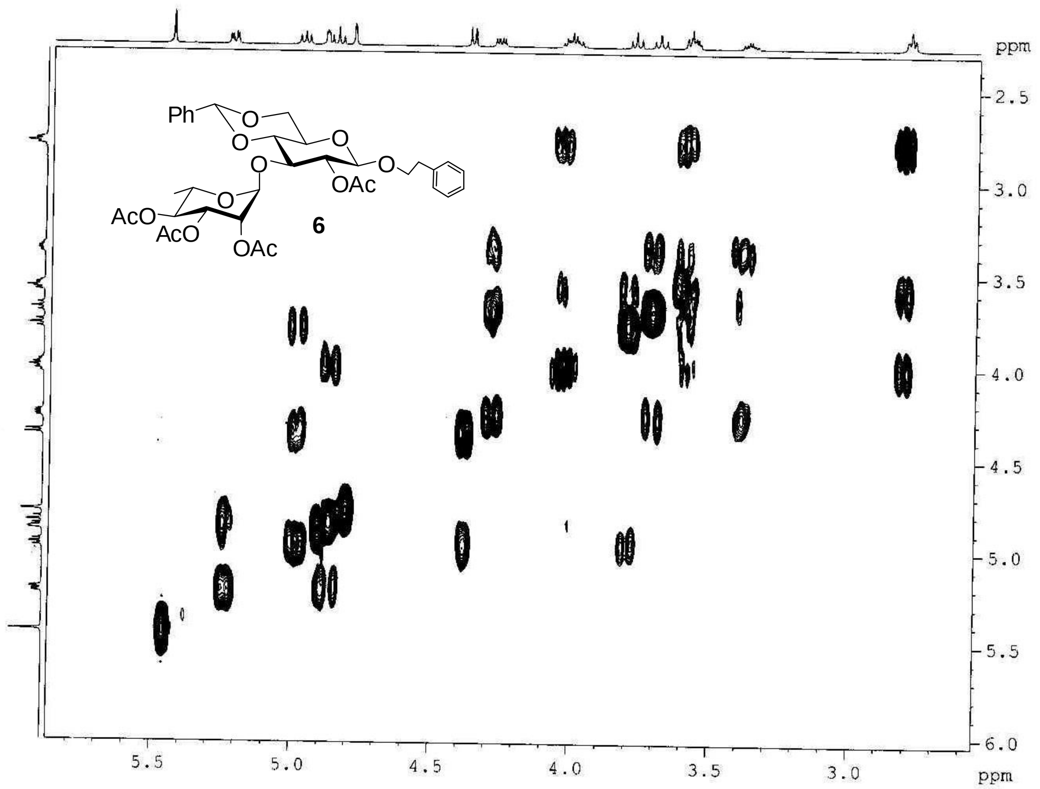

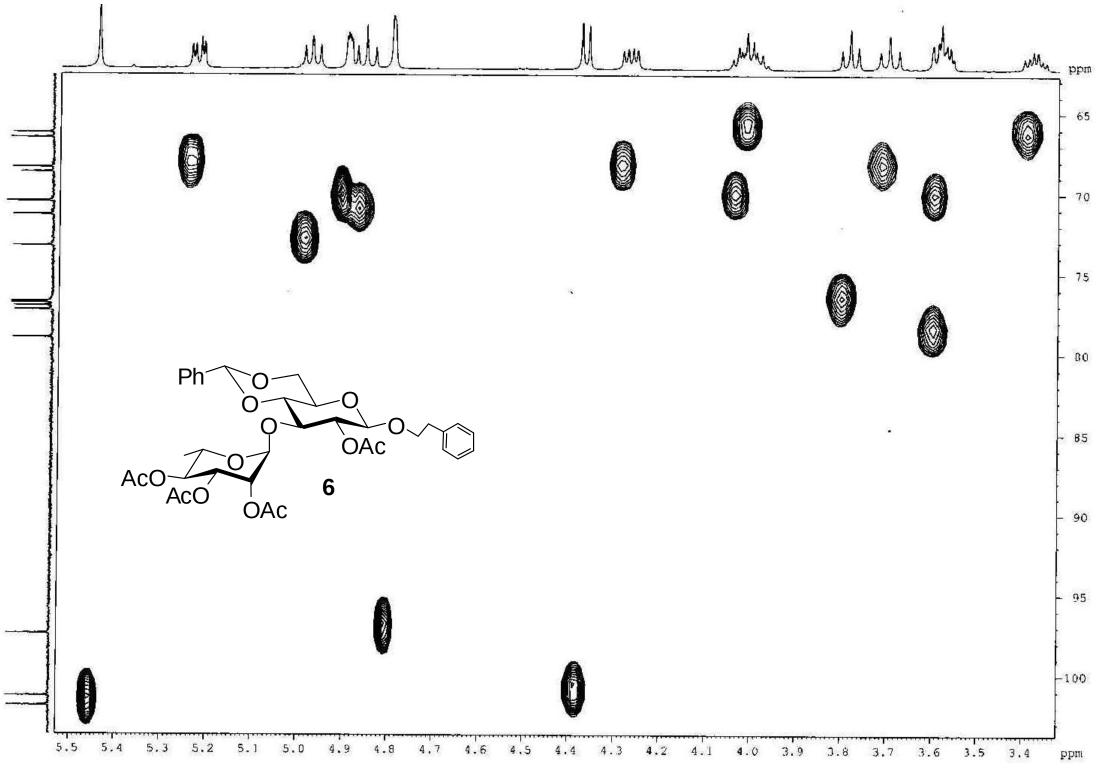

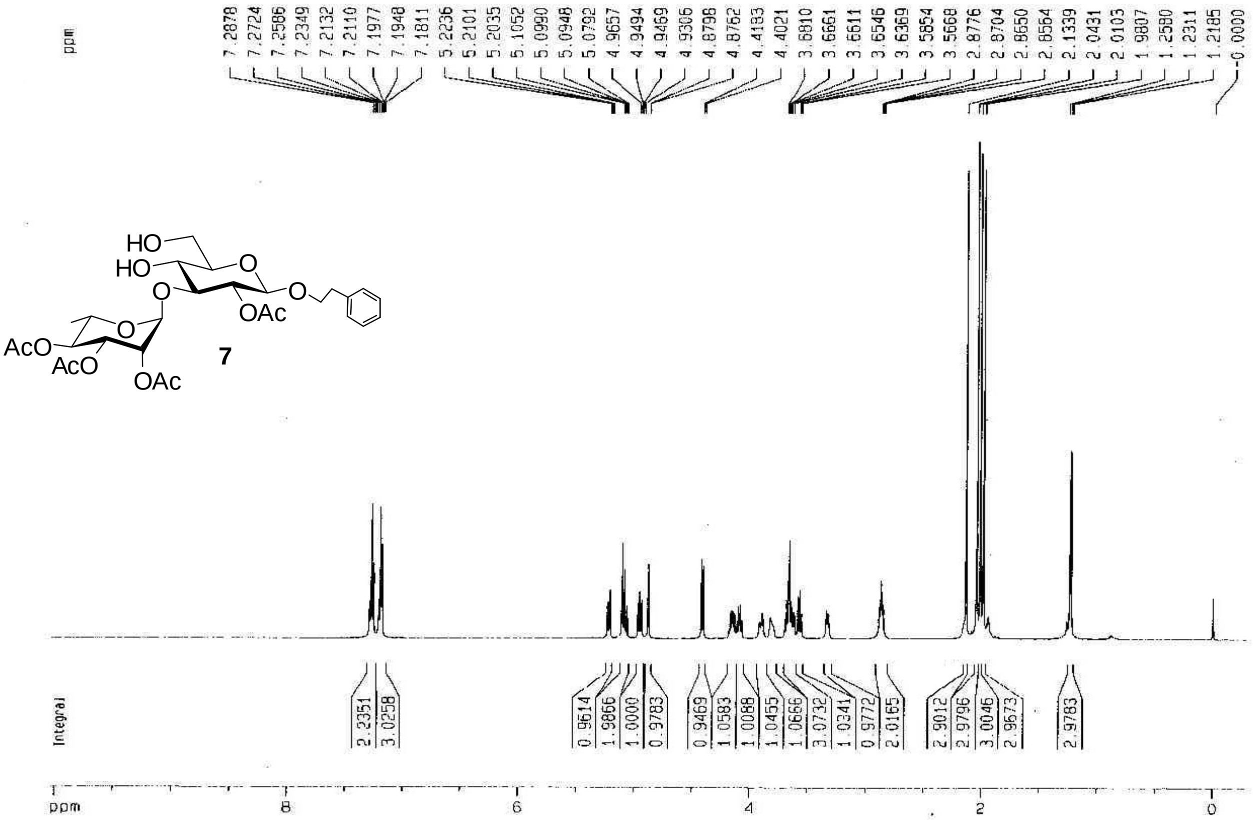

HO
HO
O
O
OAc
AcO
AcO
O
OAc
7
ppm
7.2878 7.2724 7.2586 7.2349 7.2132 7.2110 7.1977 7.1948 7.1811 5.2236 5.2101 5.2035 5.1052 5.0990 5.0948 5.0792 4.9657 4.9494 4.9469 4.9306 4.8798 4.8762 4.4183 4.4021 3.6810 3.6661 3.6611 3.6546 3.6369 3.5854 3.5668 2.8776 2.8704 2.8650 2.8564 2.1339 2.0431 2.0103 1.9807 1.2580 1.2311 1.2185 -0.0000
Integral
2.2351 3.0258 0.9614 1.9866 1.0000 0.9783 0.9469 1.0583 1.0088 1.0455 1.0666 3.0732 1.0341 0.9772 2.0165 2.9012 2.9796 3.0046 2.9673 2.9783
ppm 8 6 4 2 0

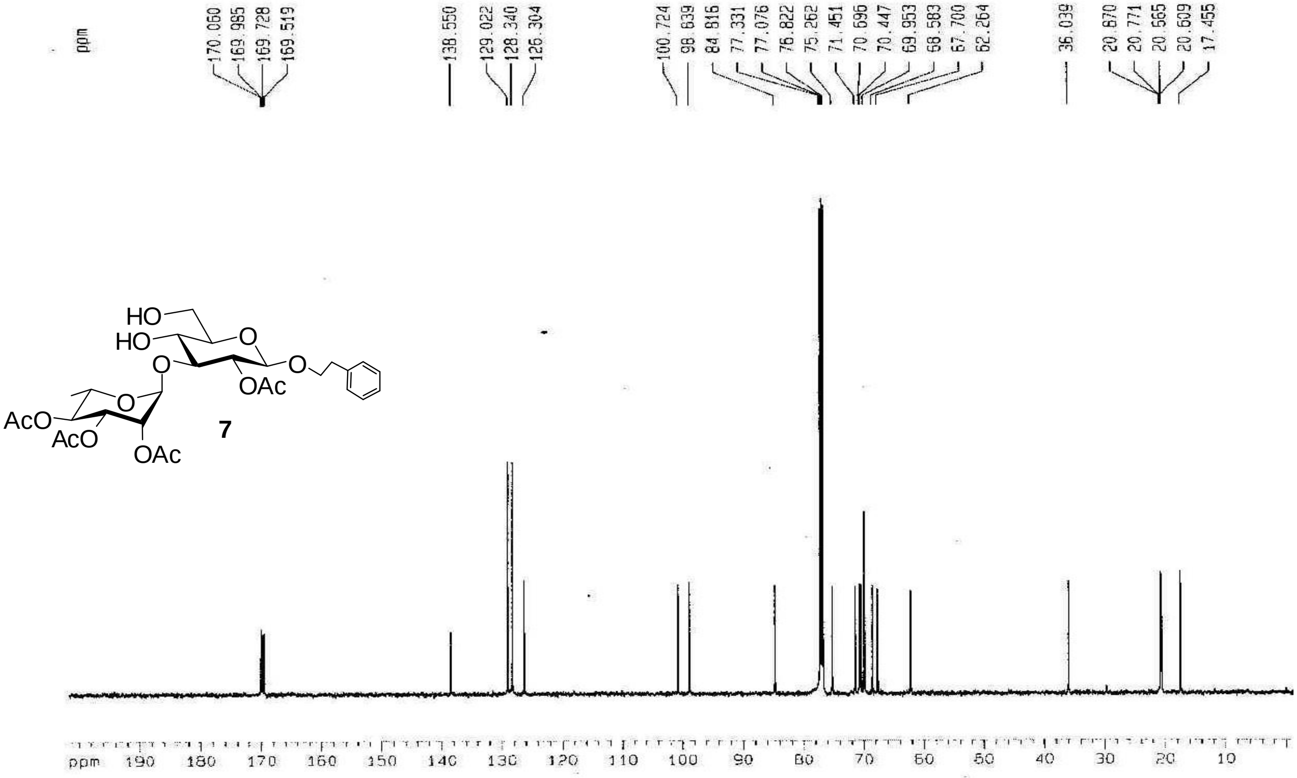

ppm
170.060 169.985 169.728 169.519 138.550 129.022 128.340 126.304 100.724 98.639 84.816 77.331 77.076 76.822 75.262 71.451 70.696 70.447 69.953 68.583 67.700 62.264 36.039 20.870 20.771 20.565 20.509 17.455
HO
HO
O
O
OAc
AcO
AcO
O
OAc
7
ppm 190 180 170 160 150 140 130 120 110 100 90 80 70 60 50 40 30 20 10

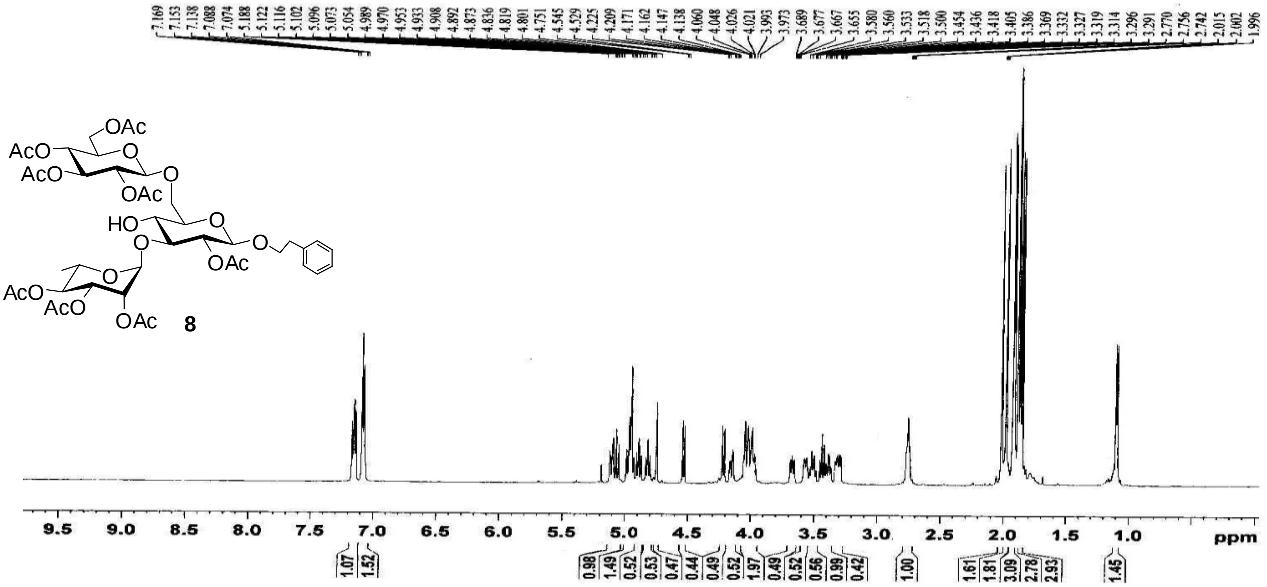
AcO
OAc
HO
O
OAc
8
9.5 9.0 8.5 8.0 7.5 7.0 6.5 6.0 5.5 5.0 4.5 4.0 3.5 3.0 2.5 2.0 1.5 1.0 ppm
1.07 1.52 0.98 1.49 0.52 0.53 0.47 0.44 0.49 0.52 1.97 0.49 0.52 0.56 0.99 0.42 1.00 1.61 1.81 3.09 2.78 2.93 1.45

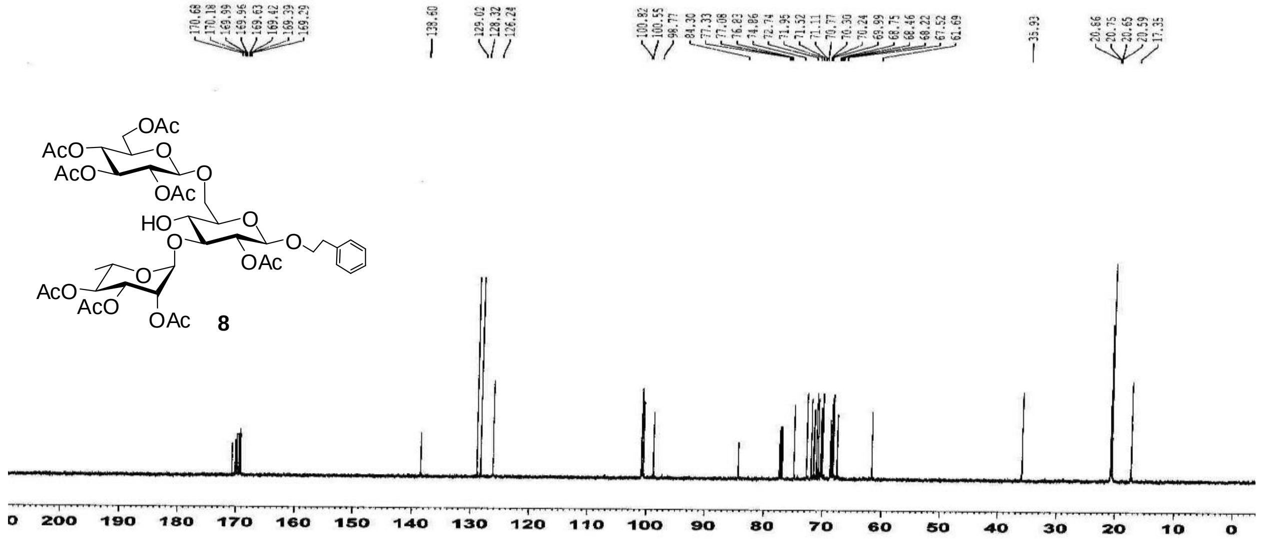
170.68 170.18 169.99 169.96 169.63 169.42 169.39 169.29
138.60
129.02 128.32 126.24
100.82 100.55 98.77
35.93
20.86 20.75 20.65 20.59 17.35
8
200 190 180 170 160 150 140 130 120 110 100 90 80 70 60 50 40 30 20 10 0

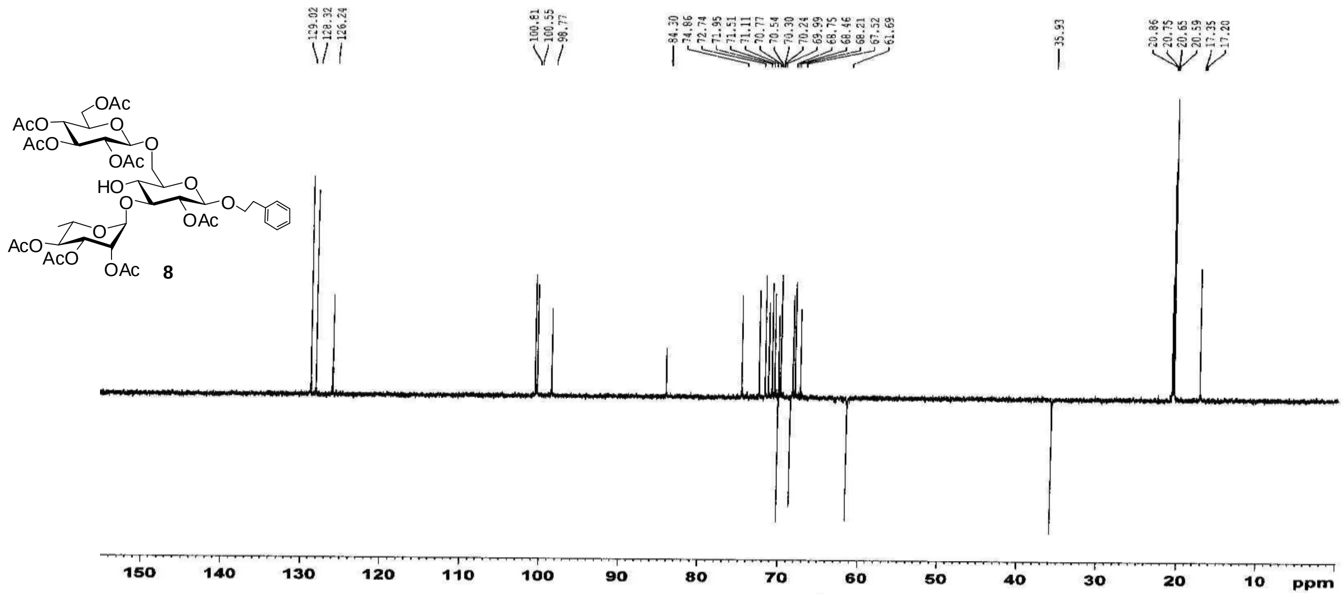
129.02 128.32 126.24
100.81 100.55 98.77
84.30
35.93
20.86 20.75 20.65 20.59 17.35 17.20
8
150 140 130 120 110 100 90 80 70 60 50 40 30 20 10 ppm

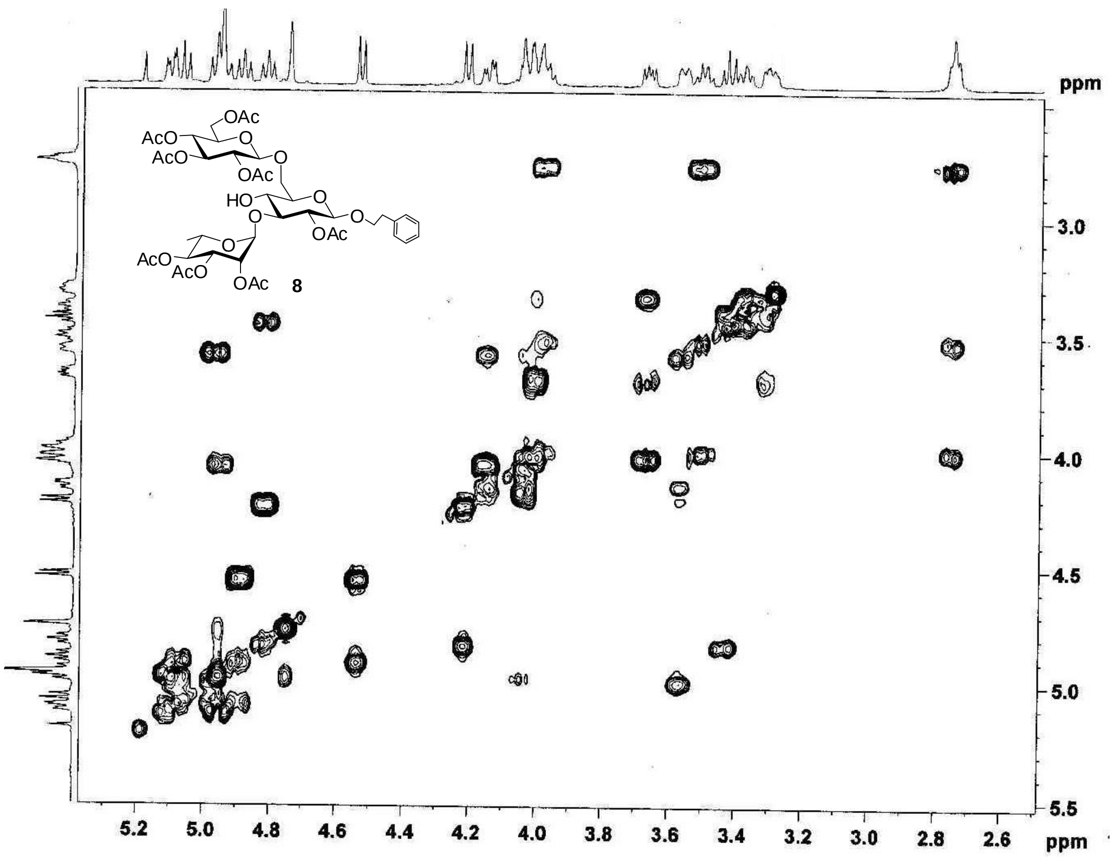
AcO
AcO
OAc
OAc
HO
O
OAc
O
AcO
AcO
OAc
8
ppm
3.0
3.5
4.0
4.5
5.0
5.5
5.2 5.0 4.8 4.6 4.4 4.2 4.0 3.8 3.6 3.4 3.2 3.0 2.8 2.6
ppm

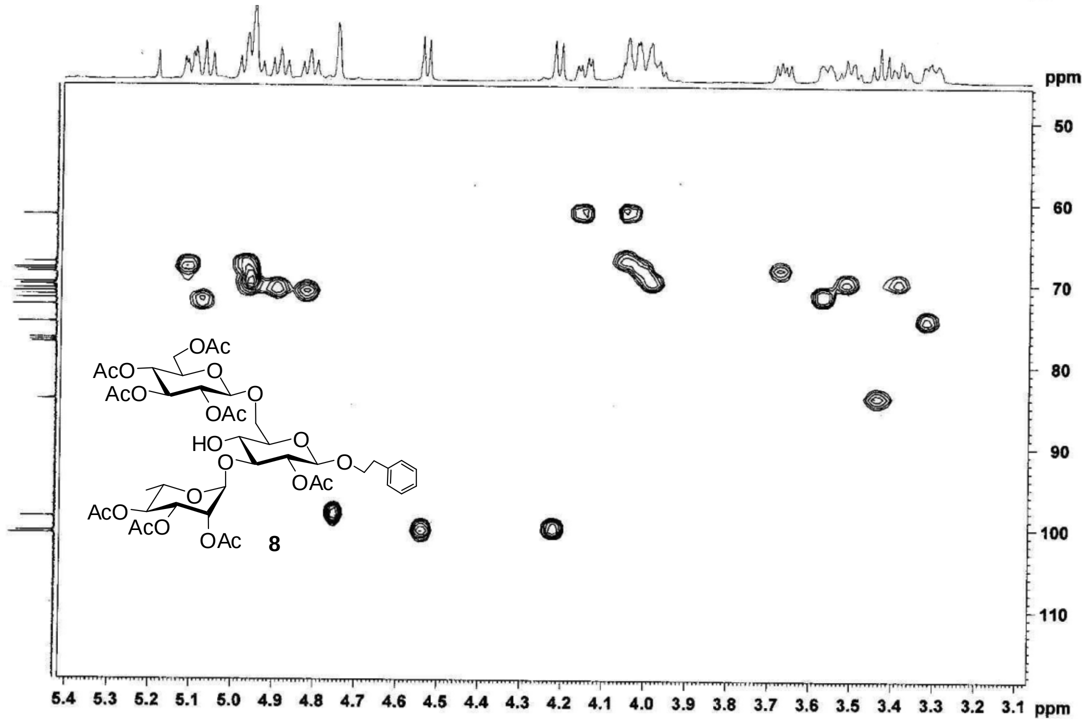
ppm
50
60
70
80
90
100
110
AcO
AcO
OAc
OAc
HO
O
OAc
O
AcO
AcO
OAc
8
5.4 5.3 5.2 5.1 5.0 4.9 4.8 4.7 4.6 4.5 4.4 4.3 4.2 4.1 4.0 3.9 3.8 3.7 3.6 3.5 3.4 3.3 3.2 3.1 ppm
